# The Pan-American Medical Congress

**Published:** 1891-11

**Authors:** 


### Correspondence.

### THE PAN-AMERICAN MEDICAL CONGRESS.

CINCINNATI, Oct. 29, a891.

*Dr. J. W. Carhart, Lampasas, Texas:*

DEAR DOCTOR:—By virtue of the power vested in me by the Committee on Permanent Organization, at St. Louis, I take pleasure in appointing you Assistant Secretary for the English countries for the Pan-American Medical Congress.

It will be your special duty to arrange with the various traffic associations for rates, counter-sign all transportation certificates, and to supervise the registration at the time of meeting; in the meantime there are various matters which may come up in which I may need to invoke your aid.

In tendering you this appointment, and in requesting that you accept it, I experience the more pleasure as it gives me an opportunity to express my appreciation of the services which you have already given the Congress, and to extend recognition not only to your great State but to one who was the very first to come forward in support of the idea of affiliating the medical profession of the western hemisphere.

Very sincerely yours,

CHARLES A. L. REED.

In reply to the above Dr. Carhart wrote as follows:

LAMPASAS, TEXAS, Nov. 6, 1891.

*Dr. Charles A. L. Reed:*

DEAR DOCTOR:—Your letter of Oct. 29th was duly received, tendering me the Assistant Secretaryship, for the English countries for the Pan-American Medical Congress, and setting forth, in part, the duties of the position.

After due deliberation, I take great pleasure in informing you that I am profoundly impressed with the dignity you have conferred upon me, and especially upon the great State of Texas in appointing me to so honorable and responsible a position in connection with a movement which I feel assured is to revolutionize, in a great measure, the quarantine and sanitary condition of the countries on this continent.

I accept, with hesitation, the position to which you have appointed me, feeling that I am scarcely able to successfully fill the place.

In accepting it, however, I assure you that whatever of ability I may possess that will in the least qualify me for so great a work, will be used to the utmost, as I desire to contribute all in my power to the fullest success of the enterprise.

Please command me to the utmost whenever I can serve you.

I am yours, respectfully,

J. W. CARHART.

